# Identifying drivers of health care value: a scoping review of the literature

**DOI:** 10.1186/s12913-022-08225-6

**Published:** 2022-06-30

**Authors:** Susan N. Landon, Jane Padikkala, Leora I. Horwitz

**Affiliations:** 1 Division of Healthcare Delivery Science, Department of Population Health, NYU Grossman School of Medicine, 227 E 30th St, Room 633, New York, NY 10016 USA; 2 Center for Healthcare Innovation and Delivery Science, NYU Langone Health, New York, NY USA; 3 Division of General Internal Medicine and Clinical Innovation, Department of Medicine, NYU Grossman School of Medicine, New York, NY USA

**Keywords:** Value, Quality, Costs, Low value care, Health services research

## Abstract

**Background:**

As health care spending reaches unsustainable levels, improving value has become an increasingly important policy priority. Relatively little research has explored factors driving value. As a first step towards filling this gap, we performed a scoping review of the literature to identify potential drivers of health care value.

**Methods:**

Searches of PubMed, Embase, Google Scholar, Policy File, and SCOPUS were conducted between February and March 2020. Empirical studies that explored associations between any range of factors and value (loosely defined as quality or outcomes relative to cost) were eligible for inclusion. We created a template in Microsoft Excel for data extraction and evaluated the quality of included articles using the Critical Appraisal Skills Programme (CASP) quality appraisal tool. Data was synthesized using narrative methods.

**Results:**

Twenty-two studies were included in analyses, of which 20 focused on low value service utilization. Independent variables represented a range of system-, hospital-, provider-, and patient-level characteristics. Although results were mixed, several consistent findings emerged. First, insurance incentive structures may affect value. For example, patients in Accountable Care Organizations had reduced rates of low value care utilization compared to patients in traditionally structured insurance plans. Second, higher intensity of care was associated with higher rates of low value care. Third, culture is likely to contribute to value. This was suggested by findings that recent medical school graduation and allopathic training were associated with reduced low value service utilization and that provider organizations had larger effects on value than did individual physicians.

**Conclusions:**

System**,** hospital**,** provider, and community characteristics influence low value care provision. To improve health care value, strategies aiming to reduce utilization of low value services and promote high value care across various levels will be essential.

**Supplementary Information:**

The online version contains supplementary material available at 10.1186/s12913-022-08225-6.

## Background

In 2018, the United States spent $3.6 trillion on health care, accounting for 17.7% of Gross Domestic Product (GDP) [1]. As a percentage of GDP, U.S. health care spending is nearly double the Organisation for Economic Cooperation and Development average, yet this does not translate into superior health outcomes [2]. Moreover, approximately 25% or more of health care spending is considered waste [3].

Researchers, health care managers, and policy makers thus have increasingly begun to focus on improving value in health care. Value is commonly conceptualized as outcomes or quality divided by costs, although the precise definition varies depending on the clinical context and stakeholder perspective. Along with this focus, large-scale initiatives to improve value have emerged. For example, the Affordable Care Act created incentives for improving quality while reducing costs through Medicare Shared Savings and bundled payment programs [4]. In addition, the American Board of Internal Medicine’s Choosing Wisely initiative generated lists of low value services across a range of specialties with the aim of reducing use of services that have little or no benefit to patients [5].

Though research on the quality and cost of care has been performed for decades, emphasis on value is more recent. Researchers seeking to understand barriers and facilitators of high value care have hypothesized that a wide range of factors—including financial incentives, delivery system structure, geography, demographics, medical education, and patient involvement—contribute to health care value and must be optimized to promote high value care [6]. In addition, extensive work has been done to identify and decrease utilization of low value services. Yet empirical studies analyzing drivers of health care value are relatively sparse. We therefore performed a scoping review to identify potential factors driving value in health care.

Understanding factors associated with health care value requires examination of drivers at multiple levels, including system-level factors (such as policies or insurance structures), hospital-level factors, physician- or practice-level factors, and patient-level factors. This review synthesizes existing literature exploring factors associated with value across these levels to begin building an understanding of how we can address disparities in value and, ultimately, create high value health care systems.

## Methods

We conducted a scoping review to identify potential drivers of value in health care. Scoping review methodology, as opposed to systematic review methodology, was selected for this study given that our aim was to provide an overview of a broad range of factors as opposed to gathering evidence in favor of a given treatment, for example [7, 8]. This review followed the five core stages of scoping reviews outlined in the methodological framework by Arksey and O’Malley [9].

### Search strategy

With the assistance of a research librarian, we searched PubMed, Embase, PolicyFile, and Google Scholar to identify articles using the terms “value,” “health care,” “low value care,” and “high value care,” in their titles. Boolean operators “AND” and “OR” were used to combine search terms where relevant. The search strategy was limited to English-language publications. No limitations were placed on publication date or study design during the search. Google Scholar hits were limited to the first 100 articles. Scopus citation tracking was also used to identify the top 20 cited articles based on the search terms “value” and “health care.” Searches were performed between February and March 2020. The complete search strategy is contained in Appendix 1. Reference lists of studies selected for inclusion after full-text screening were scanned for potentially relevant articles that may have been missed in initial searches. Grey literature was used to provide context for this review but was not used in analyses given the already broad scope of review.

### Study selection

Empirical studies that explored potential factors associated with value were eligible for inclusion. There were no limitations placed on the population studied. We defined the primary concept of interest as the value of healthcare. Only articles that used value as the dependent variable were eligible for inclusion. We accepted a broad range of definitions of value for the purposes of this review but required that the operational definition used a *combined* measure of cost and outcomes/quality or used a set of services considered to be low or high value (which implicitly includes these terms). Examples of cost measures can include expenditures for an episode of care or specific service, or extend to include downstream costs related to an initial episode of care or service. Examples of operational definitions of quality or outcomes, on the other hand, include patient experience, quality of life measures, and life-years lost or gained. Defining value based on a set of services considered to be low value relies on these services having been shown to provide little to no clinical benefit, thus creating an exceedingly high cost-benefit ratio. Studies exploring a broad range of independent variables and their associations with value were considered. No limitations were placed on context for this review, as value is important across all care settings and environments. Opinion-based articles and literature reviews were excluded from analysis. Studies that were not available in full text through NYU libraries were excluded from analysis. Studies about interventions or implementation of interventions to improve value were excluded. A table with full inclusion and exclusion criteria is presented in Appendix 2.

All studies identified through database searches were exported and uploaded into Covidence online software. Duplicates were automatically removed. An initial review of approximately 15 articles was conducted with all 3 authors to ensure agreement on inclusion criteria. One author (S.L.) then screened the remaining records by title and abstract. Potentially relevant articles were included in the full text review. Any articles of questionable eligibility were discussed by all three authors in the initial screening phase or during full text review. The final decision to include or exclude these publications was determined by consensus. Studies meeting all inclusion criteria after full-text review were kept for data extraction. Reference lists of included studies were hand-searched for any articles that may have been missed in initial searches.

### Data extraction and quality appraisal

A data extraction template was created in Microsoft Excel. Elements extracted from eligible articles included basic publication information, study design, population, definition of value (stated and/or operational), statistical methods, main results, conclusions, and limitations. The Critical Appraisal Skills Programme (CASP) quality appraisal tool for cohort studies was used to assess methodological quality of included articles [10]. All articles best fit the CASP cohort studies tool. One author (S.L.) completed all data extraction and quality appraisal.

### Data analysis

Findings from studies were synthesized using narrative methods, characterizing studies based on the independent variables (i.e. drivers of value) studied. We organized these drivers into four domains, based on the Institute of Medicine’s delivery system framework: the economic environment (insurance characteristics), the organizational environment (health system and practice characteristics), the care team (physician and other care provider characteristics) and the individual patient (patient-level characteristics) [11]. Zotero was used to manage citations for all articles included in the manuscript. Writing of the review followed PRISMA guidelines for scoping reviews [12].

## Results

### Study selection

**Fig. 1:**
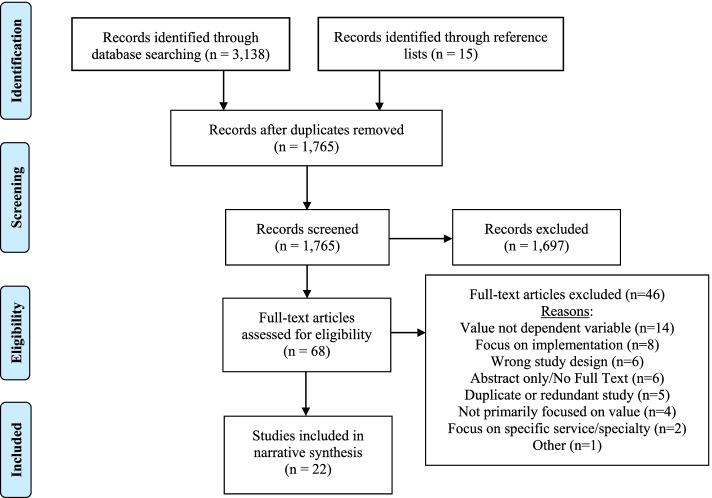
PRISMA Flow Diagram of Study Selection Process [10]

After removing duplicates, a total of 1750 publications were identified through initial searches. An additional 15 publications were identified through hand searching reference lists of articles selected for inclusion. Following screening by title and abstract, 68 were included in the full-text review. Ultimately, 22 studies were found to meet inclusion criteria. The three most common reasons for excluding articles after full text review were that the article did not use value as the dependent variable, focused on implementation of interventions to improve value, or used an ineligible study design (e.g. literature review, opinion-based article). A PRISMA flow-diagram is presented in Fig. 1 to illustrate the study selection process [13].

### Characteristics of included studies

Seventeen of the 22 studies exploring factors associated with value in health care were retrospective cohort or cross-sectional studies [14–30]. Three studies used a difference-in-difference or interrupted time series approach [31–33] and two used simulation modelling methods [34, 35]. Eighteen studies focused on low value care, two on both low and high value, [23, 29] and two on overall value [27, 35]. Among the included studies, 17 were set in the US, 3 in Canada, [16, 22, 24] 1 in England, [33] and 1 in Australia [34]. Table 1 provides a summary of the methods and major findings of included studies.

**Table 1:** Summary of Included Studies

First Author Year Country	Value Definition	Population	Independent Variable(s) and Covariates	Outcome	Major Findings (Adjusted, if presented)
**Insurance or Health System Characteristics**					
Barnett^a^, [29] 2017 US	Defined based on a set of low value services	Patient visits recorded in NAMCS and NHAMCS between 2005 and 13 and 2005–11, respectively (*n* = 193,062 eligible office visits)	Independent Variables: patient’s insurance at the time of the outpatient visit and the proportion of vulnerable patients seen by a given physician Covariates: year, age, sex, race/ethnicity, comorbidity, region, rural, practice setting	9 low value and 12 high value services identified in published guidelines and prior literature and composite measures of high and low value service utilization	Similar low and high value service use in Medicaid, uninsured, and privately insured. One exception was Medicaid and uninsured patients (compared to private) were more likely to get inappropriate narcotic prescriptions. In composite measures, Medicaid patients had lower receipt of high value services compared to privately insured (aOR = 0.88, 0.83–0.94). Uninsured had higher receipt of high value services compared to privately insured (aOR = 1.44, 1.28–1.61). No significant difference in high/low value service use between safety-net and non-safety-net physicians. Sensitivity analyses did not change results.
Braithwaite, [35] 2010 US	“Defined by the ratio of incremental benefits to incremental costs”	US Population	Independent Variables: VBID reducing cost sharing for high value services with or without increasing cost sharing for lower value services (no cost-offset, cost-offset without uninsured subsidy, cost-neutral with uninsured subsidy) Covariates: age, insurance	Life years gained, spending changes	60% of US health expenditures spent on low value services (ICER>$300,000/life year), 20% on intermediate value services (ICER $100–$300,000/life year), 20% on high value services (ICER $100,000/ life year). Applying VBID with no cost offset led to 0.24 life years gained and $22 billion increased spending. Cost offset without uninsured subsidy led to 0.25 life years gained and unchanged to reduced spending. Cost offset and uninsured subsidy led to 0.44 life years gained and no change in spending. Sensitivity analyses did not change results.
Charlesworth, [17] 2016 US	Defined as a set of services that “provide little clinical benefit and may even cause harm”	2012–13 Oregon Medicaid and commercial claims for 18–64 in Oregon All Payer All Claims Database	Independent Variable: Medicaid vs commercial insurance Covariates: age, sex, rural, comorbidities, primary care service area	13 low value services from Schwartz et al. and 3 CMS Quality Net measures	No consistent association was found between insurance type and low value care. Medicaid patients were more likely to receive low value care for services in the ED (p_interaction_ < 0.001), Medicaid patients were more likely to receive low value care if they lived in an area with a higher rate of commercial low value care for 7/11 services.
Colla, [19] 2018 US	Low value services are those whose “avoidance would increase the quality and value of care provided”	Fee for service Medicare beneficiaries enrolled in parts A and B from 2009 to 11 and commercially insured with claims in the Health Care Cost Institute claims base (*n* = 933.4 M)	Independent Variable: payer type Covariates: Hospital referral region, Medicare spending, physician group concentration, ratio of specialists to PCPs, mortality, proportion in poor or fair health, race, ethnicity, Medicare effective use score, rural, income, quality	7 low value services and a composite measure of them	Across payer types, use of low value care varied little for almost all measures. Those with commercial insurance had higher use of 3 measures of low value care. Those with Medicare had higher use of a different 3 measures of low value care. Correlation between Medicare and commercial low value care ranged from 0.540–0.905. Some regional health system characteristics (e.g. specialist to PCP ratio, Medicare spending, and physician group concentration) and patient characteristics (e.g. proportion in poor or fair health, race, poverty) were associated with low value care in both cohorts, but most of the associations were weak.
Coronini-Cronberg, [33] 2015 England	Low value procedures are those considered to be “ineffective, overused, or inappropriate”	2002–12 admissions and outpatient appointments at NHS hospitals in England recorded in HES dataset (*n* = 5,248,808 episodes)	Independent Variables: Before/after efficiency savings program, commissioning organization Covariates: year, sex, age, socioeconomic status	3 services considered ineffective and 3 services considered effective only in certain situations	Annual rate (per-1000) for each procedure varied from 0.99–3.87. Changes following implementation of the efficiency savings program varied with rates of utilization declining for 3 low value services or increasing or remaining unchanged for others. Rates of utilization of two benchmark procedures during this year were unchanged. Changes in utilization of low value services also varied considerably across commissioning organizations.
Koehlmoos, [20] 2019 US	Low value: overuse or inappropriate care, including procedures and treatments that are clinically inappropriate, excessively intensive, or too frequent	All TRICARE Prime and Prime Plus beneficiaries 18+ in 2014. This includes 20% active military, dependents, and retired military personnel (*n* = 2.9 M)	Independent variable: direct vs purchased care Covariates: age, sex, race	19 low value service indicators identified by Segal et al.	Six procedures were used more frequently in direct care settings, 11 more frequently in purchased care settings, and two showed no significant difference. Note: no adjustment for patient characteristics
Reid, [31] 2017 US	Low value “services are medical tests and procedures that provide unclear or no clinical benefit, but still expose patients to risk and expense”	25% random sample of patients 18–64 in a United health plan from 2011 to 13 not in HMOs or exclusive provider organizations (*n* = 11,074 CDHP, 365,016 traditional)	Independent Variable: CDHP vs traditional plan Covariates: age, sex, race, household income, region, comorbidities	26 measures of outpatient low value services and their associated costs. Also looked at costs for frequently co-occurring services or outpatient costs for complex services	Switching to a CDHP was associated with a $231.60 reduction (95% CI: -$341.65, −$121.53) in yearly outpatient spending. There was no significant association between switching to a CDHP and low value spending. There was a small association between switching to a CDHP and reduced low value outpatient spending on imaging (−$1.76, −$3.39, −$0.14), but no difference in low value imaging spending relative to overall imaging spending. Results remained true after sensitivity analyses.
Schwartz, [32] 2015 US	Low value services are those that “provide minimal clinical benefit on average”	Random sample of 2009–12 Medicare beneficiaries enrolled in Parts A and B (n_ACO_ = 693,218 person-yrs, n_traditional_ = 17,453,423)	Independent Variable: ACO vs traditional Medicare plan Covariates: age, sex, race, ethnicity, disability, comorbidities, hierarchical condition category risk score, geographic area	31 claims-based measures of low value care derived from evidence-based lists of low value services	In year 1 of Pioneer ACO contracts, there was a differential reduction in low value service use in the ACO group (− 0.8 services/100 beneficiaries, − 1.2 to − 0.4). This corresponded to a 4.5% (− 7.5% to − 1.5%) differential reduction in spending on low value services. Similar reductions were seen for high- and low-priced services and services that were more or less sensitive to patient preferences. ACOs with higher baseline levels of low value service use exhibited a higher differential reduction of services.
**Hospital Characteristics**					
Badgery-Parker, [34] 2019 Australia	Low-value: “tests and interventions for which the benefit is not expected to outweigh the harm and/or costs”	All patients in public hospitals in New South Wales hospitals who receive a procedure considered to be low value (*n* = 225 hospitals)	Independent Variables: Hospital Local Health District Statistical Local Area Covariates: age, comorbidities, private insurance	Receipt of any of 9 low value inpatient procedures selected from Choosing Wisely, RACP Evolve, and NICE	Hospital is a more significant contributor to low value care than local health district or statistical local area. For knee arthroscopy, the hospital MOR was 4.3 (95% CI: 3.3–5.7), meaning the odds of a patient receiving the procedure was, on average, 4.3 times higher if the patient went to a hospital in the same local health district with more low value utilization. Hospital MORs for other services were 1.7–3.5. Younger age and higher comorbidity score were associated with reduced odds of low value care.
Weeks, [27] 2016 US	Value equals quality divided by expenditures	All hospitals with available Hospital Compare and Medicare spending data for AMI, CABG, colectomy, and hip replacement in 2012	Independent Variables: Hospital Size Hospital Census Number of Beds Accreditation Hospital Profit Status	Value as defined by a composite measure of patient experience, quality of care processes, outcomes, and safety divided by expenditures for the episode of care	Hospitals in highest value quintiles had higher census (*p* < 0.003), more beds (*p* < 0.019), and more operations (*p* < 0.001 for 2/4 conditions) compared to those in the lowest value quintile. Accreditation was not associated with value. CABG did not show the same associations with hospital census, beds, operations, and accreditation. Only hip replacement was associated with hospital profit status (*p* < 0.006). Half of hospitals in highest or lowest value quintiles also in highest or lowest quality quintile.
**Physician/Practice Characteristics**					
Barreto, [15] 2019 US	Low value: “unnecessary services” or “‘overuse beyond evidence based levels and unnecessary choice of higher cost services”’	Stratified random sample of PCPs matched to 2011 Medicare Part B beneficiaries (*n* = 6873 PCPs, 1,078,840 patients)	Independent Variables: PCP sex, years in practice, specialty, credential, international graduate status, patient panel size, location Covariates: age, sex,race, ethnicity, comorbidities	Cost of primary care services considered low value based on Choosing Wisely recommendations	Lower annual per patient low value care Medicare spending was associated with allopathic training (β = −$1.65), smaller Medicare patient panel (β = −$3.98 for panels < 50 vs panels > 300), family medicine practice (β = −$1.03 vs internal medicine), practicing in the Midwest (β = −$2.80 vs Northeast), practicing in a rural area (β = −$1.75), and being a recent graduate.
Bouck, [16] 2018 Canada	Low value care “represents little to no patient benefit, or comparatively greater risk of harm”	Identified test-specific cohorts across Ontario outpatient visits for 4 low value tests. Excluded physicians with < 50 in 2012–14. (*n* = 2394 physicians)	Independent Variables: physician sex, international graduate status, years since graduation, billing group care model and size Covariates: patient sociodemographic variables	4 screening tests identified as low value based on Choosing Wisely Canada recommendations	Physicians who were male (OR = 1.29, 1.01–1.64)), further out from medical school graduation (OR = 1.03, 1.02–1.04), domestic graduates (OR = 1.56, 1.19–2.04), or in an enhanced fee for service payment model (OR = 2.04, 1.42–2.94 vs capitated) had increased odds of being frequent users of low value tests. 18.4% of physicians ordered 39.2% of low value tests. Billing group differences of greater relevance than patient or physician factors.
Mafi, [21] 2016 US	Low value care is care that often has a “greater probability of harm than benefit”	Patients with primary care visits recorded in NAMCS or NHAMCS from 1997 to 2011 for 3 conditions. (*n* = 25,529 physician and 3420 APC visits)	Independent Variable: APC vs MD Covariates: Age, sex, race/ethnicity, comorbidities, symptom acuity, insurance status, rural, region, year, PCP vs not PCP, hospital vs community practice, practice setting type	Low value services identified for each of these conditions based on Choosing Wisely recommendations	APCs were not significantly more likely to order antibiotics (*p* = 0.96 office-based, *p* = 0.92 hospital-based), CT/MRI (*p* = 0.086 office-based, *p* = 0.48 hospital-based) or radiography (*p* = 0.65 office-based, *p* = 0.73 hospital-based), or refer to specialist physicians compared to physicians (*p* = − 0.64 office-based, *p* = 0.091 hospital-based). When stratified by PCP vs non-PCP, hospital-based PCP APCs ordered more antibiotics and made more referrals than hospital-based PCP physicians.
Mafi, [14] 2017 US	Low value care is “care that provides minimal average benefit in specific clinical scenarios”	Patients with primary care visits recorded in NAMCS or NHAMCS from 1997 to 2013 for URTI, back pain, or headache. (*n* = 31,162)	Independent Variables: hospital/community-based practice, hospital or physician owned, Covariates: age, sex, race/ethnicity, usual PCP, comorbidities, insurance status, rural, region, year, symptom acuity	Low value services identified for each of these conditions based on Choosing Wisely recommendations	Visits to hospital-based practices had higher use of CT or MRI (OR = 1.44, 1.13–1.85), radiographs (1.41, 1.16–1.71), and specialty referrals (2.74, 2.23–3.36) compared to community based. Antibiotic use was similar in both locations. Non-PCP visits associated with higher use of imaging and specialty referral, primarily in hospital practices. No significant differences in hospital owned vs physician owned practice in terms of antibiotic and imaging use, but hospital owned had more specialty referrals.
Oronce, [23] 2019 US	High Value: “guideline-concordant care that improves health, avoids harm, eliminates waste;” Low value: overuse of “services that provide marginal or unknown benefit”	All visits from patients 18+ to PCPs with eligible ICD9 codes recorded in NAMCS in 2010–12. (*n* = 29,155)	Independent Variables: Community health center vs private practice Covariates: age, sex, race/ethnicity, insurance, comorbidities, rural, region, year	12 measures of high value care and 7 measures of low value care consistent with previously published guidelines and studies	Community health centers were more likely to prescribe beta blockers for CHF visits (OR = 2.56, 1.18–5.56), statins for diabetes (OR = 1.35, 1.02–1.79), and treatment for osteoporosis (OR = 1.77, 1.05–3.00) compared to private practices. They were also more likely to avoid screening urinalysis (OR = 1.87, 1.11–3.14), complete blood count (OR = 1.72, 1.18–2.53), and EKGs (OR = 11.03, 2.67–45.52), and they were less likely to prescribe antibiotics for upper respiratory infections (OR = 0.59, 0.40–0.88). Overall, community health centers performed better or comparably to private practices on high and low value care measures explored in this study.
Schwartz, [30] 2018 US	Low value services “provide minimal average clinical benefit in specific clinical scenarios”	20% sample of 2007–11 Medicare FFS patients in parts A and B matched to provider organizations (*n* = 4,039,733 patients and 3137 provider organizations)	Independent variable: provider organization defined by TIN Covariates: age, sex, race/ethnicity, disability, ESRD, comorbidities, area-level socioeconomic status	Rate of utilization of 31 low value services based on previous studies and guidelines as well as a composite measure of low value service utilization	Between organization standard deviation in use of low value services were 10 (95% CI: 9.4–40.5). Variation in services more sensitive to patient preference (90th:10th percentile = 1.61, 1.58–1.64) was much less than variation in services categorized as less sensitive (90th:10th percentile = 2.84, 2.6–3.13). Within organizations, service use was positively correlated between almost all pairs of categories of low value services.
Schwartz, [28] 2019 US	Low value services are those that “produce minimal average clinical benefit in specific clinical scenarios”	20% sample of Medicare FFS beneficiaries enrolled in parts A and B from 2008 to 13 matched to PCPs (*n* = 3,159,834 beneficiaries and 41,773 PCPs)	Independent variables: physician age, sex, training, academic engagement, payment from pharmaceutical or device manufacturer, patient panel size Covariates: Patient age, sex, race/ethnicity, disability, dual eligibility, ESRD, area-level SES	Rate of use of 17 low-value services (based on previous studies and guidelines) reported as number of services per 100 patients per year	Mean rate of low value service provision was 33.1 services per 100 beneficiaries per year. Smaller variation in rates of low value service utilization observed than predicted by differences in physician characteristics. Within organizations, ratio of low value service use between providers at the 90th and 10th percentiles of low value use was 1.67 per 100 beneficiaries per year. Variation in low value service utilization across organizations was much greater than would be expected due to chance. The only physician characteristic predicting substantially higher low value service utilization was patient panel size.
**Physician and Patient Characteristics**					
Colla, [18] 2014 US	Low value defined by “services whose avoidance improve efficiency through higher quality, reduced risks, and lower costs”	Fee for service Medicare beneficiaries enrolled in Parts A and B from 2006 to 11	Variables: Hospital referral region, per-beneficiary Medicare spending, physician group concentration, ratio of specialists to PCPs, quality, mortality, percent in poor or fair health, race, ethnicity, effective use score, rural, income	11 services representing 37 Choosing Wisely recommendations that can be measured using Medicare claims data	Higher Medicare spending was associated with low value care utilization (coeff = 0.099, 0.053–0.145) as was higher ratio of specialists to PCPs (coeff = 0.343, 0.060–0.626), higher proportion of black (coeff = 0.018, 0.011–0.025) and Hispanic (coeff = 0.015, 0.001–0.023), and higher proportion of residents in poor/fair health (coeff = 0.026, 0.001–0.052). Higher poverty rate (coeff = − 0.025, − 0.044, − 0.006) and higher physician group concentration (coeff = − 0.008, − 0.148, − 0.002) was associated with lower low value care utilization. Quality was not associated with low value service use.
McAlister, [22] 2017 Canada	Low value care is defined as “health care practices that provide minimal or no benefit to recipients”	All patients 18+ in Alberta that presented to a health care provider between 2012 and 15 (n = 3,423,135)	Independent Variables: age, sex, comorbidities, region, physician contacts in past 12 months, median household income, specialist to PCP ratio	10 low value services identified from Choosing Wisely lists that have been evaluated before and can be identified using claims data	Higher socioeconomic status (OR = 1.14–1.46 for each of 4 low value services), increased frequency of specialist contact (OR = 1.003–1.006 for each of 4 low value services), and higher ratio of specialists to PCPs (OR = 1.22–7.79 for each of 4 low value services) were associated with increased likelihood of receiving low value services. Provision of low value services varied significantly across eight regions, but not consistently in one direction.
**Patient Characteristics**					
Pendrith, [24] 2017 Canada	Low value care is care with a lack of benefit that can lead to higher health care costs and inconvenience or harm to patients	Ontario patients meeting eligibility criteria as recorded in the OHIP claims database between 2008 and 2013 (*n* = 9,918,145)	Variables: age, sex, rural, neighborhood income quintile, practice, Local Health Integration Network	3 Choosing Wisely recommendations relevant to primary care	Substantial variation in all services, with repeat DEXA scan being the most common (21% of those receiving an index scan) and low value cervical cancer screening being the only one to decrease significantly in the study period. Predictors of repeat DEXA scan include older (*p* < 0.001), female (p < 0.001), urban (p < 0.001), high income area (p < 0.001), and high risk index scan (p < 0.001). Note: results not adjusted
Reid, [25] 2016 US	Defined based on a set of low value services	25% random sample of 2011–13 Optum Clinformatics Data Mart claims for United Healthcare patients 18–64 across the US (*n* = 1,468,689)	Variables: age, sex, race, ethnicity, income, area, Consumer-Directed Health Plan	28 low value care measures previously published. Assessed number of patients receiving each service and associated costs	Low value spending per $10,000 in total spending was less among patients who were older (35–49 yrs.: − 20.42 [− 22.03, − 18.81]; 50–64 years: − 11.30 [− 12.94, − 9.66] compared to 18–34), male (− 18.19 [− 19.34, − 17.03]), black (− 3.81 [− 5.68, − 1.95]), Asian (− 4.40 [− 7.23, − 1.57]), low-income (− 8.10 [− 10.55, − 5.66]), or enrolled in a Consumer Directed Health Plan (− 5.86 [− 7.51, − 4.21]). West South Central region had highest effect of low value spending per $10,000 (14.26 [12.17, 16.35]).
Schpero, [26] 2017 US	Low value services are those which are “unnecessary and economically inefficient, many are potentially harmful”	2006–11 Medicare beneficiaries at risk of receiving a specified low value service (n varied by test)	Independent Variables: Race, ethnicity Covariates: age, income, sex, disability status, Medicaid enrollment, risk score, health care utilization, year, hospital referral region	11 low value services identified by Choosing Wisely	Black and Hispanic beneficiaries had qualitatively higher receipt of low value services compared to white beneficiaries before adjusting. After adjustments, receipt of low value care was significantly higher among blacks for 5/11 services and for 6/11 services for Hispanics.

*ACO* Accountable Care Organization, *AMI* Acute Myocardial Infarction, *aOR* Adjusted Odds Ratio, *APC* Advanced Practice Clinician, *CABG* Coronary Artery Bypass Graft, *CDHP* Consumer Directed Health Plan, *CHF* Congestive Heart Failure, *CT* Computed Tomography, *DEXA* Dual Energy X-Ray Absorptiometry, *ED* Emergency Department, *EKG* Electrocardiogram, *ESRD* End Stage Renal Disease, *FFS* Fee for Service, *HES* Hospital Episode Statistics, *HMO* Health Maintenance Organization, *ICER* Incremental Cost Effectiveness Ratio, *MD* Medical Doctor, *MOR* Median Odds Ratio, *MRI* Magnetic Resonance Imaging, *NAMCS* National Ambulatory Medical Care Surveys, *NHAMCS* National Hospital Ambulatory Medical Care Surveys, *NICE* National Institute for Health and Care Excellence, *OHIP* Ontario Health Insurance Plan, *OR* Odds Ratio, *PCP* Primary Care Provider, *RACP* Royal Australasian College of Physicians, *SES* Socioeconomic Status, *TIN* Taxpayer Identification Number, *URTI* Upper Respiratory Tract Infection, *VBID* Value-Based Insurance Design

^a^Looks at insurance and provider characteristics

The 20 studies that explored factors associated with low value used receipt of low value services, as identified using claims data, as the outcome variables. Of these 20 studies, three also looked at costs associated with the specified low value services as an outcome [15, 25, 31]. In contrast to those studies, Weeks et al., invented a composite measure of value to use as the primary outcome variable. Defining value generally as quality divided by expenditures, the authors deconstructed quality into four component pieces which can be summarized as patient experience, care processes, care outcomes, and safety [27]. Data for each of these components was drawn from Hospital Compare. The authors standardized these measures, multiplied the component pieces together, then divided by total expenditures for the episode of care in order to get a measure of value [27]. On the other hand, Braithwaite et al., defined value as incremental benefits over incremental costs and examined incremental changes in life expectancy and costs as the outcome variables [35].

### Quality appraisal

Overall, the quality of included studies was high (Appendix 3). All studies had clearly stated objectives. All of the low value studies used large, representative datasets for cohort selection, and claims-based measures to assess receipt of low value services. Weeks et al. used Hospital Compare data, which allowed for examination of a broader definition of value but lacked some granularity given its aggregation of data [27]. Braithwaite et al. used a validated computer simulation of the entire US health care system [35].

Two articles considered to be of lower quality were those by Koehlmoos et al., and Pendrith et al., as these studies did not adjust for potential confounding variables [20, 24]. The remaining 20 studies adjusted for potential confounders and/or conducted sensitivity analyses. In general, most studies included identified patient-level characteristics as the main potential confounders.

In terms of generalizability of the studies, given the data sources and study designs, it is likely that the findings would apply to the local populations studied. It is unclear, however, if a subset of services considered to be low value is representative of all low value care, and thus if the results are generalizable to all low value care. It is also unclear if results would be applicable to different populations or health systems.

### Insurance and health system characteristics

Six studies examined associations between insurance characteristics and receipt of low value services [17, 19, 20, 29, 31, 32]. One study examined associations between different value-based insurance designs and incremental changes in life expectancy and cost [35]. One study examined effects of a national efficiency savings program on low value service utilization [33]. Of the studies exploring associations between insurance characteristics and low value service utilization, five studies showed no clear pattern of significant associations between insurance type and low value care [17, 19, 20, 29, 31]. Three of these studies compared low value service utilization in commercial versus public insurance, [17, 19, 29] one compared direct versus purchased care, [20] and one compared those in a Consumer Directed Health Plan versus traditional plan [31]. Barnett et al. found similar rates of low value care in Medicaid, uninsured, and privately uninsured populations [29]. However, they also found that Medicaid patients were less likely than privately insured patients to receive high value services when pooling estimates of high value service utilization whereas uninsured were more likely to receive high value services compared to their privately insured counterparts. The authors speculated that the latter finding may have been due to unmeasured confounding in a highly select population [29].

In contrast to studies that found no associations between insurance type and value, Schwartz et al. found that patients enrolled in a Pioneer Accountable Care Organization (ACO) Medicare plan saw a differential reduction in the use of low value service (− 0.8 services/100 beneficiaries; 95% CI: − 1.2,-0.4) in the first year of the contract compared to those who remained on a traditional Medicare plan [32]. Accountable Care Organizations are groups of healthcare providers that jointly contract with an insurer (typically, Medicare) to provide coordinated care in exchange for a share of any savings. Some also share in any excess costs. In a computer simulation study, Braithwaite et al. found that value-based insurance designs reducing cost-sharing for high value services and increasing cost-sharing for low value services were associated with a 0.24–0.44 gain in life years without increasing spending [35]. Finally, Coronini-Cronberg et al. found mixed effects on low value service utilization across commissioning organizations from a national efficiency savings policy applied to the National Health Service [33].

### Hospital characteristics

Two studies included focused on hospital characteristics associated with value [27, 34]. Badgery-Parker et al. used a multilevel modelling design to test the effects of hospital and region on receipt of low value care. They found that hospital is a more significant contributor to low value care than region [34]. Weeks et al. looked at hospital characteristics such as census, beds, number of operations, accreditation, and for-profit status. They found that higher value was associated with higher census, more beds, and more operations [27].

### Physician and practice characteristics

Seven studies focused on physician and/or practice characteristics [14–16, 21, 23, 28, 30]. Two additional studies explored physician characteristics in addition to patient characteristics, [18, 22] and one study explored physician characteristics in addition to insurance characteristics [29]. Physician characteristics associated with reduced use of low value services include more recent medical school graduation, allopathic training, smaller patient panel, and being female [15, 16]. Mafi et al. also found that Advanced Practice Clinicians (APCs) had comparable low value service utilization to physicians [21] and Barnett et al. found that safety-net and non-safety net providers had comparable low value service utilization [29].

Investigating both physician and practice variation in low value service utilization, Schwartz et al. found that physician characteristics account for relatively little variation in provision of low value services while organizations contribute substantially more to variation in low value service provision [28] Another study by Schwartz et al. demonstrated a standard deviation of 10 low value services per 100 beneficiaries between provider organizations, further supporting that low value service utilization varies substantially across provider organizations [30]. Other articles identified more specific practice characteristics that may contribute to variation in rates of low value service utilization. For example, fee-for-service practices had higher rates of low value service utilization than capitated practices, [16] community health centers had higher rates of high value care and lower rates of low value care than private practices, [23] and hospital-based practices had higher use of low value services than community-based practices [14]. In addition, two studies showed higher specialist to primary care provider ratios were associated with greater low value care utilization [18, 22].

### Patient characteristics

Three studies explored patient characteristics associated with low value care [24–26] in addition to the two studies that explored both physician and patient characteristics [18, 22].. Two studies found that being female was associated with higher rates of low value care utilization compared to being male [24, 25]. Two studies showed that black and Hispanic patients had received low value services more frequently [18, 26]. In contrast, Reid et al. found decreased low value spending among black beneficiaries [25]. Four studies showed that higher income was associated with increased rates of low value care utilization [18, 22, 24, 25]. Three studies showed that region was associated with low value care utilization, although patterns for this varied [22, 24, 25].

## Discussion

In this review examining factors associated with health care value, we identified 22 studies exploring a range of system- (including insurance), hospital-, provider-, and patient-level characteristics and their associations with value. Across the 22 studies included, almost all [20] focused on utilization of low value services as the dependent variable. Although included studies showed mixed results, several more consistent findings emerged, many of which are reminiscent of findings from past research on factors associated with health care quality and factors associated with health care spending.

One overarching finding from studies included in this review is that incentive schemes may be important drivers of low value service utilization, but only when structured certain ways. For example, in the included articles, neither consumer-directed health plans nor public vs private insurance were associated with low value service utilization, but ACO enrollment was associated with reduced utilization of low value services and value-based insurance design was predicted to increase life expectancy while keeping expenditures constant [32, 35]. ACOs are designed with a primary aim of improving value, and these findings suggest that such a structure may in fact be helpful in doing so. ACO and Alternative Quality Contract enrollment has also been shown to be associated with reduced spending and higher quality, although associations with quality have been less consistently shown [36–39].

This review also illustrated considerable variation in value by region, hospital, and provider type [22, 24, 25, 27, 28, 30, 34]. Similarly, previously published research has shown regional variation in quality and spending [40–42] as well as variation in readmission rates by hospital [43]. In many cases, the associations found with value were similar to those found with quality. For instance, much like Weeks et al. found that higher volume hospitals tended to have higher value, Birkmeyer et al. showed that higher hospital volume was associated with lower surgical mortality—an important element of hospital quality [44]. However, it is important to note that value and quality are distinct. Weeks et al., for instance, found that 50% of the hospitals in the highest or lowest value quintiles were not in the highest or lowest quality quintiles, respectively [27].

Additionally, this review underscored that greater intensity of care (defined as higher ratio of specialists to primary care providers, hospital-based, and fee-for-service payment structure) was associated with higher utilization of low value services [14, 16, 18, 22]. This trend is similarly reflected in quality and cost literature. For example, Wennberg et al. found that greater intensity of hospital care was associated with lower quality care and lower patient experience ratings [45]. In terms of cost, Barnett et al., found that hospital-based physician networks were associated with higher costs and higher intensity of care [46].

Studies included in this review also highlighted physician characteristics associated with value, showing that recent medical school graduation and allopathic training was associated with lower utilization of low value services [16, 18]. It is plausible that this finding reflects changing medical school cultures at allopathic institutions, which are now placing a greater emphasis on value. Tsugawa et al. found that patients treated by older physicians had higher 30-day mortality rates compared to those treated by younger physicians; this difference was not observed in high volume providers nor when comparing readmission rates [47]. They also found slightly higher costs in older physicians [47]. In contrast, Mehrotra et al. found that more recent medical school graduates tended to have higher cost profiles [48]. This discrepancy could reflect several factors including change over time in medical education, lack of concordance between overall spending and spending on low value services, or, given that one of the articles on physician characteristics associated with low value service utilization was set in Canada, there could also be differences across countries. Additionally, Schwartz et al. found that provider organizations had much larger effects on low value service utilization than individual physician characteristics, providing further support for the role of value culture and potentially explaining some of this uncertainty [28].

Higher income and female sex were associated with lower value care in several studies included in this review. Interestingly, opposite results have been found with studies of quality and cost individually. Okunrintemi et al. found that low income patients reported lower quality of care, greater difficulty accessing care, and worse experiences of care compared to high income patients [49]. Epstein et al. also found that patients of a lower socioeconomic status had longer hospital stays and higher charges compared to those with a higher socioeconomic status [50]. Bird et al. showed that women tended to receive higher quality of care than men except in areas related to cardiovascular disease and adverse drug-disease interactions [51]. These differences again highlight the difference between low value service utilization and provision of high quality care.

Importantly, studies included in this review overwhelmingly focused on low value, highlighting current limitations in data and metrics and raising the question of whether patterns of low value service utilization are reflective of value more generally. Although reducing utilization of low value services may seem to be a logical approach to improving value, it is likely insufficient to create a high value health care system. As described in this review, there was a paradoxical finding that high volume was associated with high value, whereas greater intensity of care was associated with higher utilization of low value services. This seeming contradiction provides evidence of a rift between what is deemed high value (including excellent patient outcomes) and the mere absence of low value service utilization. Moreover, studies have repeatedly found that efforts to influence utilization tend to change rates of both appropriate and inappropriate care [52, 53].

By contrast, only four articles in this review explored factors associated with high value (or value) in health care, possibly because high value is often conflated with high quality. In fact, Oronce et al. and Barnett et al. operationalized high value using lists of services that are frequently considered to be quality process measures. But high value and high quality are not the same construct. A health system providing high quality care at very high cost is likely not high value; a system providing both high quality care and excessive low value services is also not high value. These tradeoffs are explicitly addressed in cost effectiveness literature, but this review illustrates that they have had limited use to date when identifying “high value” care.

### Strengths and limitations

This scoping review provides an overview of the literature exploring factors associated with value in health care. It is the first to bring together this body of literature as a step towards understanding and utilizing the complex interplay of system-, hospital-, physician-, and patient-level characteristics driving value.

This review has several limitations which are important to address. First, as a scoping review, this relied on title searches to identify relevant articles. Thus, it is possible that relevant studies were missed given the constraints of the search. However, we searched reference lists of included studies to identify additional articles. Second, studies included explored a range of factors that may affect value, with value having been defined using different lists of low value services and different levels of sensitivity and specificity. Consequently, study findings did not elucidate a consistent factor driving value, and comparability and implications of results must be interpreted with caution. Third, given that included studies were primarily observational, we cannot establish causality. Fourth, given the heterogeneity of studies included, it was not possible to conduct a meta-analysis or combine the findings in any quantitative manner. Finally, although three researchers were involved in the planning of the study and any questions that arose regarding inclusion and exclusion criteria, only one reviewer selected articles for inclusion and appraised the quality, making it prone to researcher bias. Pre-determined criteria were used for article selection and standardized templates were used for data extraction in an effort to mitigate this bias, but we acknowledge that this is nonetheless a major limitation of this study.

## Conclusion

A complex interplay of system-, hospital-, provider-, and patient-level factors appear to be driving value in health care. In this review, we found evidence supporting the role of insurance incentive schemes, intensity of care, and culture as key drivers of health care value. Although there exists some overlap between factors driving quality of care and factors driving health care value, we found they are not identical, and thus the two constructs must be considered as distinct entities. Moreover, drivers of high value care and drivers of low value care appear to differ. This is especially important to consider when developing interventions to improve value in health care and establishing standards for defining and measuring value. To effectively improve health care value, developing interventions to address drivers across multiple levels of care as well as novel measurement strategies to capture both high and low value practices will be essential.

## Supplementary Information

**Additional file 1: Appendix 1.** Search Strategy. **Appendix 2.** Full Set of Inclusion and Exclusion Criteria.

**Additional file 2: Appendix 3.** Summary of Quality Appraisal.

## Data Availability

Not applicable; no additional data available.
