# Supplementary material for: Identifying drivers of health care value: a scoping review of the literature
Source: BMC Health Serv Res. 2022 Jun 30;22:845. doi: 10.1186/s12913-022-08225-6 (PMC9248090; doi:10.1186/s12913-022-08225-6)
Supplement: Supplementary file 1 — Additional file 1: Appendix 1. Search Strategy. Appendix 2. Full Set of Inclusion and Exclusion Criteria. [file 12913_2022_8225_MOESM1_ESM.pdf]

Appendix 1. Search Strategy

	Search	Filter	Date	Hits	Exported
Scopus					
	TITLE (value AND (healthcare OR "health care"))	English	2.10.20	1,710	20 (Top Cited)
PubMed					
	low value care[Titl]	English	2.11.20	85	85
	("value"[Titl]) AND ("health care"[Titl] OR "healthcare"[Titl])	English	2.28.20	1,064	1,064
	high value care[Titl]	English	3.12.20	122	122
Embase					
1	Value.m_titl.		3.23.20	128.288	
2	Healthcare.m_titl.		3.23.20	56,234	
3	Health care.m_title.		3.23.20	100,869	
4	2 or 3		3.23.20	156,821	
5	1 and 4		3.23.20	1,296	
6	Limit 5 to English	English	3.23.20	1,241	1,241
7	High value care.m_titl.	English	3.23.20	160	160
8	Low value care.m_title.	English	3.23.20	95	95
Policy File					
	ti(value) AND (ti(health care) OR ti(health care))	English	3.25.20	47	47
	ti(high value care)	English	3.25.20	4	4
	ti(low value care)	English	3.25.20	0	0
Google Scholar					
	allintitle: value "health care" OR healthcare	English	3.25.20	3,960	100
	allintitle: high value care	English	3.25.20	531	100
	allintitle: low value care	English	3.25.20	306	100

Appendix 2. Full Set of Inclusion and Exclusion Criteria

Inclusion	Exclusion
Original research looking at factors associated with value	Abstracts
English language	Articles introducing a journal issue
	Replies
	Protocols
	Focused on implementation of practices or interventions to improve value
	Opinion-based article
	Articles focused on a specific service or specialty
	Literature Review
