# Supplementary material for: Identifying drivers of health care value: a scoping review of the literature
Source: BMC Health Serv Res. 2022 Jun 30;22:845. doi: 10.1186/s12913-022-08225-6 (PMC9248090; doi:10.1186/s12913-022-08225-6)
Supplement: Supplementary file 2 — Additional file 2: Appendix 3. Summary of Quality Appraisal. [file 12913_2022_8225_MOESM2_ESM.pdf]

	Did the study address a clearly focused issue?	Was the cohort recruited in an acceptable way?	Was the exposure accurately measured?	Was the outcome accurately measured?	Have the authors identified all important confounding factors?	Have they taken account of the confounding factors in the design and/or analysis?	Was the follow-up. Of subjects complete?	Was the follow-up of subjects long enough?	How precise are the results?	Do you believe the results?	Can the results be applied to the local US population?*	Do the results of this study fit with other available evidence?	What are the implications of this study for practice?
Badgery-Parker, 2019											*Based in NSW*		
Barnett, 2017											*Based in US*		
Barreto, 2019											*Based in US*		
Bouck, 2018											*Based in Ontario*		
Braithwaite, 2010											*Based in US*		
Charlesworth, 2016											*Based in Oregon*		
Colla, 2014											*Based in US*		
Colla, 2018											*Based in US*		
Cronberg, 2015											*Based in England*		
Koehlmoos, 2019											*Based in US*		
Mafi, 2016											*Based in US*		
Mafi, 2017											*Based in US*		
McAlister, 2017											*Based in Alberta*		
Oronce, 2019											*Based in US*		
Pendrith, 2017											*Based in Ontario*		
Reid, 2016											*Based in US*		
Reid, 2017											*Based in US*		
Schpero, 2017											*Based in US*		
Schwartz, 2015											*Based in US*		
Schwartz, 2018											*Based in US*		
Schwartz, 2019											*Based in US*		
Weeks, 2016											*Based in US*		

*Note: Setting was considered when addressing generalizability in data synthesis

KEY
Yes/Completely
Mostly
Partially/Can't Tell
No/Not at All
